# Supplementary material for: Porcine Reproductive and Respiratory Syndrome (PRRS) and CD163 Resistance Polymorphic Markers: What Is the Scenario in Naturally Infected Pig Livestock in Central Italy?
Source: Animals (Basel). 2023 Jul 31;13(15):2477. doi: 10.3390/ani13152477 (PMC10417267; doi:10.3390/ani13152477)
Supplement: Supplementary file 1 [file animals-13-02477-s001.zip › Figure S1.pdf]

Figure S1: Pairwise linkage disequilibrium (LD) plot for the variants in *CD163* gene, expressed as D′ values represented within the matrix.

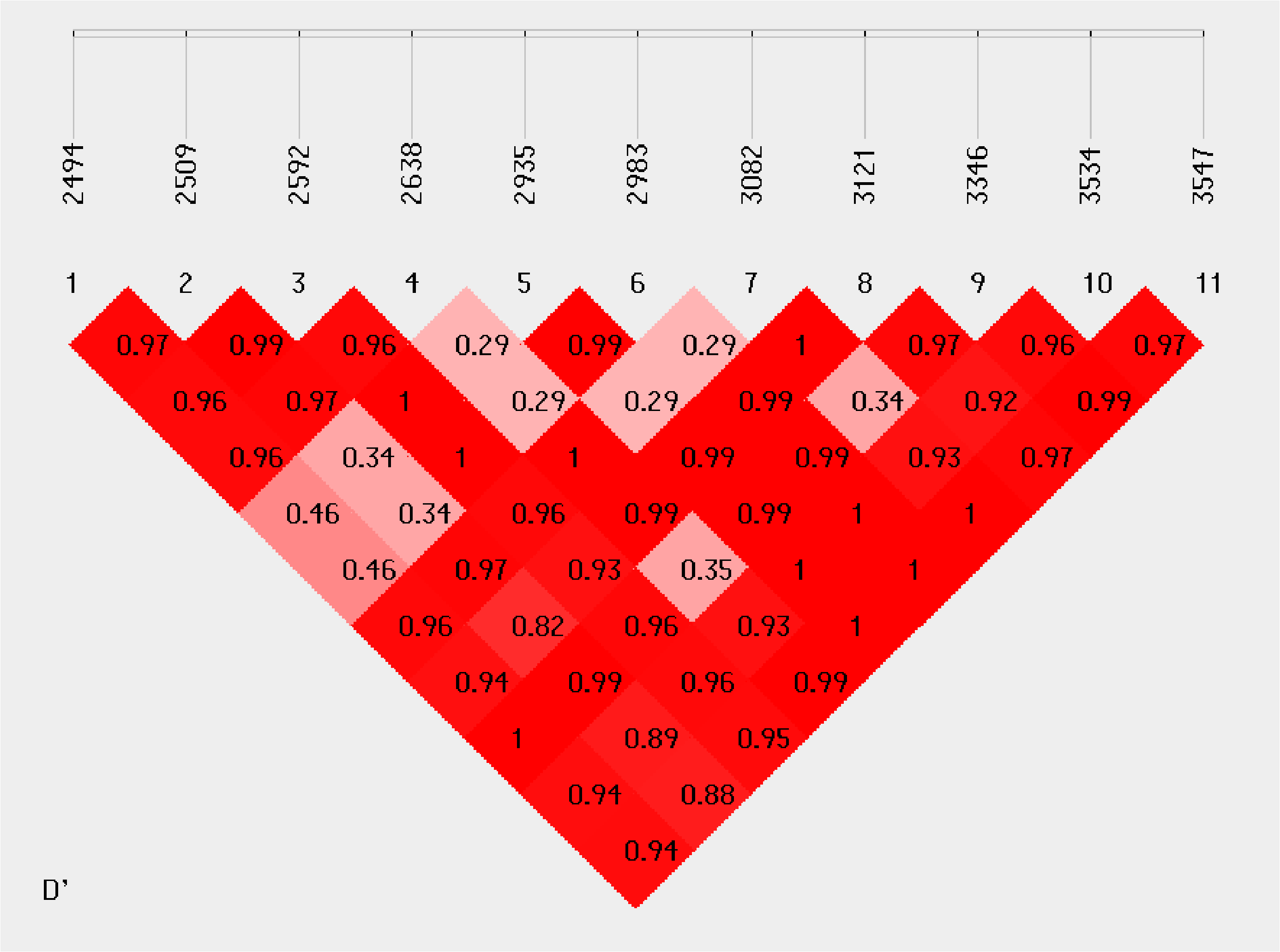

Dark red boxes indicate strong LD; lighter red boxes indicate progressively lesser LD
