# Supplementary material for: Porcine Reproductive and Respiratory Syndrome (PRRS) and CD163 Resistance Polymorphic Markers: What Is the Scenario in Naturally Infected Pig Livestock in Central Italy?
Source: Animals (Basel). 2023 Jul 31;13(15):2477. doi: 10.3390/ani13152477 (PMC10417267; doi:10.3390/ani13152477)
Supplement: Supplementary file 1 [file animals-13-02477-s001.zip › Table S1.pdf]

Table S1: Frequencies (%) of haplotypes and genotypes reconstructed by Phase v 2.1 software

N	Haplotype	N	Freq. (%)	Genotype	Healthy	Infected	Total	Freq. (%)
**1**	GGAGGCCTGCA	6	0,80	(16,16)	50	15	65	17,2
**2**	GGAGGCCTGTA	17	2,25	(16,30)	57	6	63	16,7
**3**	GGAGGCCTACA	120	15,92	(3,9)	46	1	47	12,5
**4**	GGAGGCCTATA	2	0,27	(3,16)	38		38	10,1
**5**	GGAGGCCCGCG	6	0,80	(9,30)	29	3	32	8,5
**6**	GGAGGCCCACA	1	0,13	(3,30)	14	6	20	5,3
**7**	GGAGGCTTGCA	1	0,13	(30,30)	18	1	19	5,03
**8**	GGAGAACCGCG	1	0,13	(9,16)	16		16	4,2
**9**	GGAAGCTTGCA	103	13,66	(2,30)	6		6	1,6
**10**	GGAAGCTTGTA	6	0,80	(16,19)	4		4	1,06
**11**	GGGGGCCTGCG	1	0,13	(3,3)	3		3	0,8
**12**	GGGGGCCTACA	1	0,13	(1,20)		2	2	0,53
**13**	GGGGGCCCGCG	7	0,93	(1,30)	2		2	0,53
**14**	GGGAGCTTGCA	1	0,13	(10,32)	2		2	0,53
**15**	GCAGGCCTGCA	1	0,13	(13,16)	2		2	0,53
**16**	GCAGGCCTGTA	265	35,15	(19,30)	1	1	2	0,53
**17**	GCAGGCCTACA	1	0,13	(2,2)	2		2	0,53
**18**	GCAGGCCTATA	1	0,13	(2,3)	1	1	2	0,53
**19**	GCAGGCCCGCG	7	0,93	(22,30)	2		2	0,53
**20**	GCAGGCCCGCA	3	0,40	(27,30)	2		2	0,53
**21**	GCAGGCCCGTA	8	1,06	(3,21)	2		2	0,53
**22**	GCAGAACCGCG	3	0,40	(5,16)	2		2	0,53
**23**	GCAAGCTTGCA	1	0,13	(9,13)	2		2	0,53
**24**	GCGGGCCCGCG	1	0,13	(3,10)	2		2	0,53
**25**	AGAGGCCCGCG	2	0,27	(13,21)	1		1	0,26
**26**	AGAAGCTTGCA	1	0,13	(16,20)	1		1	0,26
**27**	AGGGGCCTGCG	3	0,40	(16,25)	1		1	0,26
**28**	AGGGGCCTGTA	2	0,27	(16,27)	1		1	0,26
**29**	AGGGGCCTACA	1	0,13	(16,29)	1		1	0,26
**30**	AGGGGCCCGCG	174	23,08	(16,33)		1	1	0,26
**31**	AGGGGCCCGCA	1	0,13	(2,16)	1		1	0,26
**32**	AGGGGCCCGTG	2	0,27	(2,21)	1		1	0,26
**33**	AGGGGCTCGCG	1	0,13	(2,28)	1		1	0,26
**34**	AGGGAACCGCG	1	0,13	(2,8)	1		1	0,26
**35**	ACAGGCCTGTA	1	0,13	(21,21)	1		1	0,26
**36**	ACAGGCCCGCA	1	0,13	(21,30)	1		1	0,26
				(22,23)		1	1	0,26
				(24,9)	1		1	0,26
				(26,30)	1		1	0,26
				(3,28)	1		1	0,26
				(30,36)		1	1	0,26
				(4,10)	1		1	0,26
				(4,16)	1		1	0,26
				(5,10)	1		1	0,26
				(5,5)	1		1	0,26
				(5,9)	1		1	0,26

N	Haplotype	N	Freq. (%)	Genotype	Healthy	Infected	Total	Freq. (%)
				(7,30)	1		1	0,26
				(9,21)	1		1	0,26
				(9,25)	1		1	0,26
				(9,34)		1	1	0,26
				(16,35)	1		1	0,26
				(12,14)	1		1	0,26
				(11,16)	1		1	0,26
				(1,1)	1		1	0,26
				(2,13)	1		1	0,26
				(9,18)	1		1	0,26
				(6,30)	1		1	0,26
				(15,30)	1		1	0,26
				(17,30)	1		1	0,26
				(3,13)	1		1	0,26
				(3,19)	1		1	0,26
				(16,31)	1		1	0,26
